# Cadmium-induced impairment of spermatozoa development by reducing exosomal-MVBs secretion: a novel pathway

**DOI:** 10.18632/aging.204675

**Published:** 2023-04-25

**Authors:** Waseem Ali, Yusheng Bian, Hina Ali, Jian Sun, Jiaqiao Zhu, Yonggang Ma, Zongping Liu, Hui Zou

**Affiliations:** 1 College of Veterinary Medicine, Yangzhou University, Yangzhou 225009, Jiangsu, P.R. China; 2 Jiangsu Co-Innovation Center for Prevention and Control of Important Animal Infectious Diseases and Zoonoses, Yangzhou 225009, Jiangsu, P.R China; 3 Joint International Research Laboratory of Agriculture and Agri-Product Safety of the Ministry of Education of China, Yangzhou University, Yangzhou 225009, Jiangsu, P.R China; 4 University of Health Sciences, Lahore 54651, Punjab, Pakistan

**Keywords:** cadmium, nano-scale exosome, MVBs, spermatozoa

## Abstract

Cadmium is a heavy environmental pollutant that presents a high risk to male-fertility and targets the different cellular and steroidogenic supporting germ cells networks during spermatogenesis. However, the mechanism accounting for its toxicity in multivesicular bodies (MVBs) biogenesis, and exosomal secretion associated with spermatozoa remains obscure. In the current study, the light and electron microscopy revealed that, the Sertoli cells perform a dynamic role with secretion of well-developed early endosomes (Ee) and MVBs pathway associated with spermatozoa during spermatogenesis. In addition, some apical blebs containing nano-scale exosomes located on the cell surface and after fragmentation nano-scale exosomes were directly linked with spermatozoa in the luminal compartment of seminiferous tubules, indicating normal spermatogenesis. Controversially, the cadmium treated group showed limited and deformed spermatozoa with damaging acromion process and mid-peace, and the cytoplasmic vacuolization of spermatids. After cadmium treatment, there is very limited biogenesis of MVBs inside the cytoplasm of Sertoli cells, and no obvious secretions of nano-scale exosomes interacted with spermatozoa. Interestingly, the cadmium treated group demonstrated relatively higher formation of autophagosomes and autolysosome, and the autophagosomes were enveloped by MVBs that later formed the amphisome which degraded by lysosomes, indicating the hypo-spermatogenesis. Moreover, cadmium declined the exosomal protein cluster of differentiation (CD63) and increased the autophagy-related proteins microtubule-associated light chain (LC3), sequestosome 1 (P62) and lysosomal-associated membrane protein 2 (LAMP2) expression level were confirmed by Western blotting. These results provide rich information regarding how cadmium is capable of triggering impaired spermatozoa development during spermatogenesis by reduction of MVBs pathway through high activation of autophagic pathway. This study explores the toxicant effect of cadmium on nano-scale exosomes secretion interacting with spermatozoa.

## INTRODUCTION

Spermatogenesis is characterized by the mitotic phase, meiotic phase and differentiate haploid phase within the seminiferous tubules and occurs in association with and supported by the somatic Sertoli cells [1]. During this journey, the spermatozoa encounter different biological fluids secreted by Sertoli cells and interstitial cells. These complex fluids interact with the spermatozoa surface and modify the composition of macromolecules of the male gamete. These sequential modifications are essential for the production, maturation and protection of a fully functional spermatozoa. Many organs of the male reproductive tract are known to secrete membranous particles using apocrine secretions [2].

Exosomes are nanosized vesicles (30–100 nm diameter), that are actively secreted by almost all types of cells, including endothelial cells, fibroblasts, epithelial cells, immune cells, neuronal cells, as well as cancer cells [3, 4]. Similar to other biological fluids, exosomes are an endosomal pathway vesicle, that releases after multivesicular bodies (MVBs) fuse with the lysosome and plasma membrane. These vesicles are enriched with many bio-active molecules, such as nucleic acids, proteins, lipids, and metabolites, exosomes are endowed with the ability to relay signals between cells [5, 6]. Exosomes serve as early diagnostic tools for many diseases related to aging and cancer [7–9]. Currently, the influence of exosomes on the male reproductive system, and in particular their influence on the development of gametes, is of great interest [10]. Exosomes promote reproductive success through supporting spermatozoa development and function. Exosomes-MVBs pathways contribute to maintain the normal homeostasis of spermatozoa during spermatogenesis [1]. Proteomic analysis of exosomes from seminal fluids indicates the number of proteins that are involved in spermatozoa motility, capacitation or acrosomal reaction, prevent premature spermatozoa capacitation, and influence the process of fertilization due to the fact that they also carry cholesterol and sphingomyelin [11–15]. Collecting proofs now support vital roles for exosomes in cellular communication and molecular transport with extensive animal health implications linked to development of spermatozoa [16, 17]. However, how exposure of environmental toxicant modifies exosomal-MVBs pathway remains obscure.

Cadmium is the most widespread global environmental pollutant, which exerts various toxic effects in many tissues and organs of humans and animals [18, 19]. The human population is exposed to cadmium toxicity through air, food and the water [20]. The main sources of cadmium include agricultural and industrial pollution [21]. Cadmium toxicity is dependent on its biological characteristics, and it is defined as non-biodegradable with an extensive biological half-life, particularly below the earth [22]. It has previously been reported that exposure to low doses of cadmium predominantly affects the testes, and no other organs [23]. Cadmium is a strong testicular toxicant that affects different physiological processes in the testicular tissue of different animals [24, 25]. However, there has not yet been a report of the toxicant effect of cadmium on the immunological mechanism of exosomal secretion. Cadmium is considered as one of the most reproductive toxicants in males. Subsequently, the majority of studies have focused on the cadmium contributing a negative impact on the different testicular bio-chemical functions and testicular immune-related secretions, like a male gamete secretion and steroidogenic secretion of the testis, that represent a high risk factor for male fertility [26–31]. Researchers have revealed that cadmium interrupts the movement of the sperm-specific cation (KSper) and (CatSper) channels that cause male infertility [32], cadmium-induced oxidative stress through Nrf2 signaling pathway, and cadmium causes apoptosis through the p38 MAPK pathway, as well as cadmium induces high activated autophagy which causes testicular injury [33, 34]. These findings are clearly linked to a declining trend in male fertility though different pathways. However, our current study found for the first time the toxicant effect of cadmium on exosomes-MVBs secretion pathway, which may lead to destruction of spermatozoa during spermatogenesis. In addition, it will be fundamental to map out the key signaling pathways that toxicants target to modulate secretion of nano-scale exosomes.

## RESULTS

**Figure 1:**
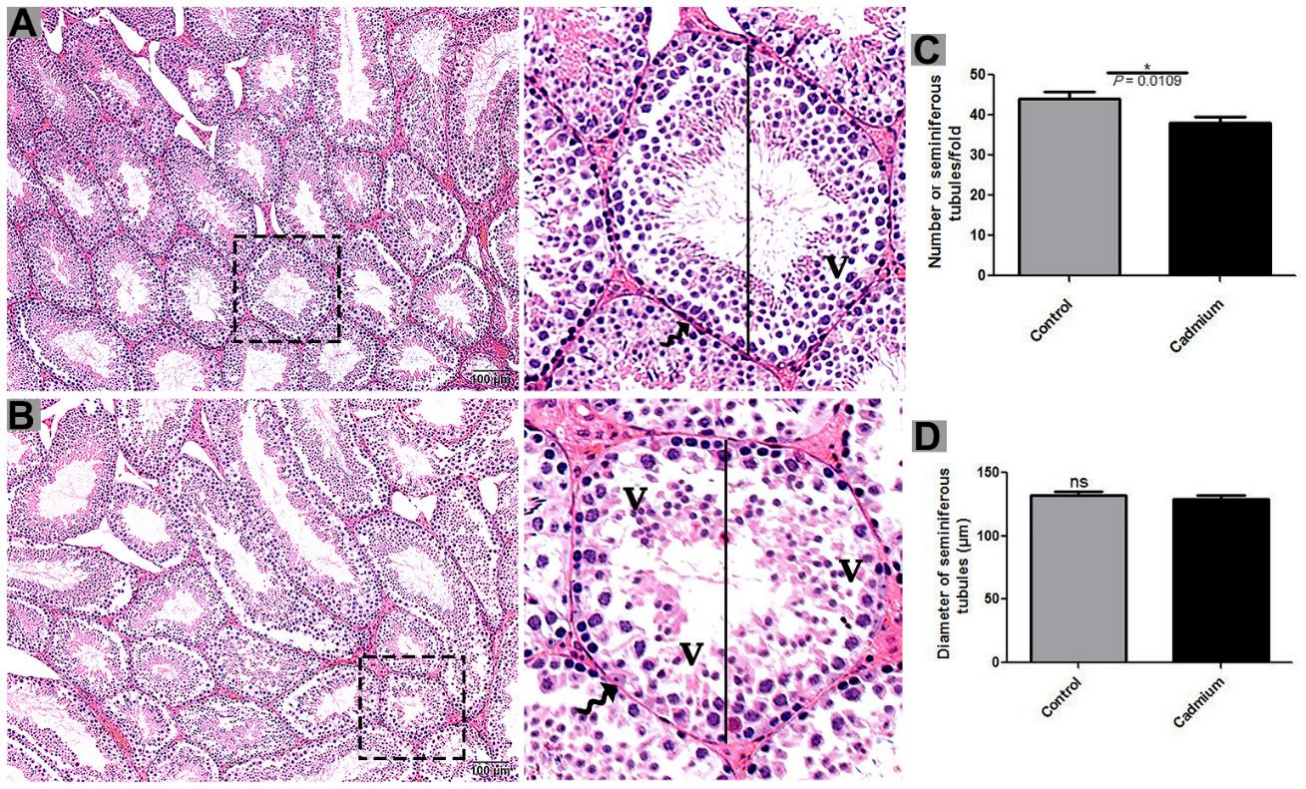
Light microscopy of seminiferous tubules of testis of control (**A**) and cadmium treated group (**B**). A large magnification is illustrated in the rectangular area. (**C**) Numbers of seminiferous tubules. (**D**) Quantification of the diameter of seminiferous tubules. V: vacuole; (curved arrow) basement membrane. Scale bars = 100 μm.

**Figure 2:**
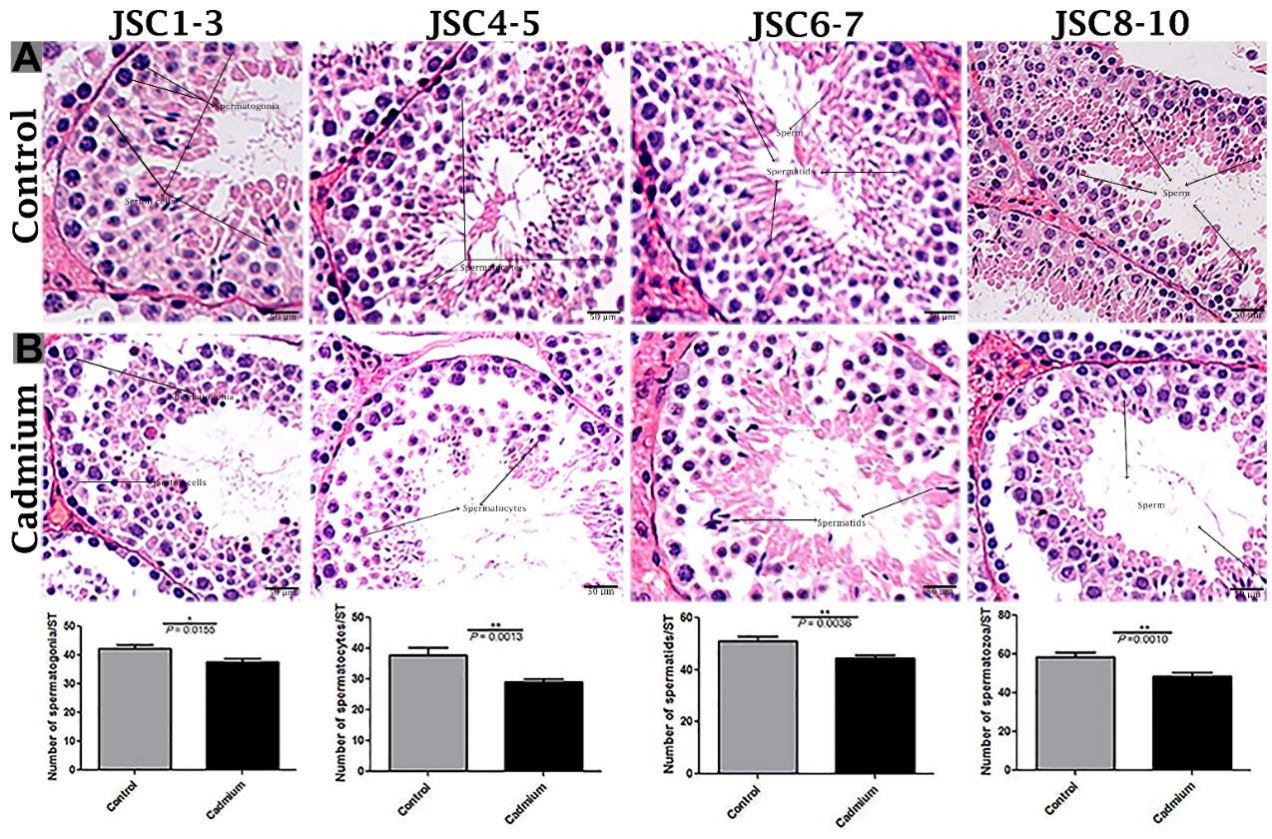
Light microscopy of seminiferous tubules of testis of control (**A**) and cadmium treated group (**B**). JSCs 1-2 lack germ cells, while JSC 3 contains spermatogonia but no spermatocytes. JSCs 4-5 contain a small number of spermatocytes but no spermatids. JSCs 6-7 contain few or many spermatids but no sperm. JSCs 8-10 contain a small or large number of sperms in a seminiferous tubule. Scale bars = 50 μm. Data presented as Mean ± SEM.

According to histological analysis, the control group showed the Sertoli cells were characterized by a compact morphology, and the more developing germ cells and permanent Sertoli cells were lined at the seminiferous tubules of testis. Furthermore, a significantly higher number of seminiferous tubules were observed in control group compared to the cadmium group without a change in seminiferous tubules diameter (Figure 1A). Controversially, in cadmium treated group, the luminal compartment of seminiferous tubules showed significantly reduced number of developing spermatogonia, spermatocytes, spermatids and spermatozoa. Additionally, disorganization of seminiferous tubules with extensive degenerative vacuolation and empty intercellular space between the germ cells were observed (Figures 1B, 2A, 2B). The above listed criteria were calculated through the Johnsen score. Johnsen score was highest in the control group, indicating normal spermatogenesis and germ cells development, whereas the contrary was the case in the cadmium group, indicating hypo-spermatogenesis.

### Cadmium-induced ultrastructural modifications in MVBs in the seminiferous tubules

**Figure 3:**
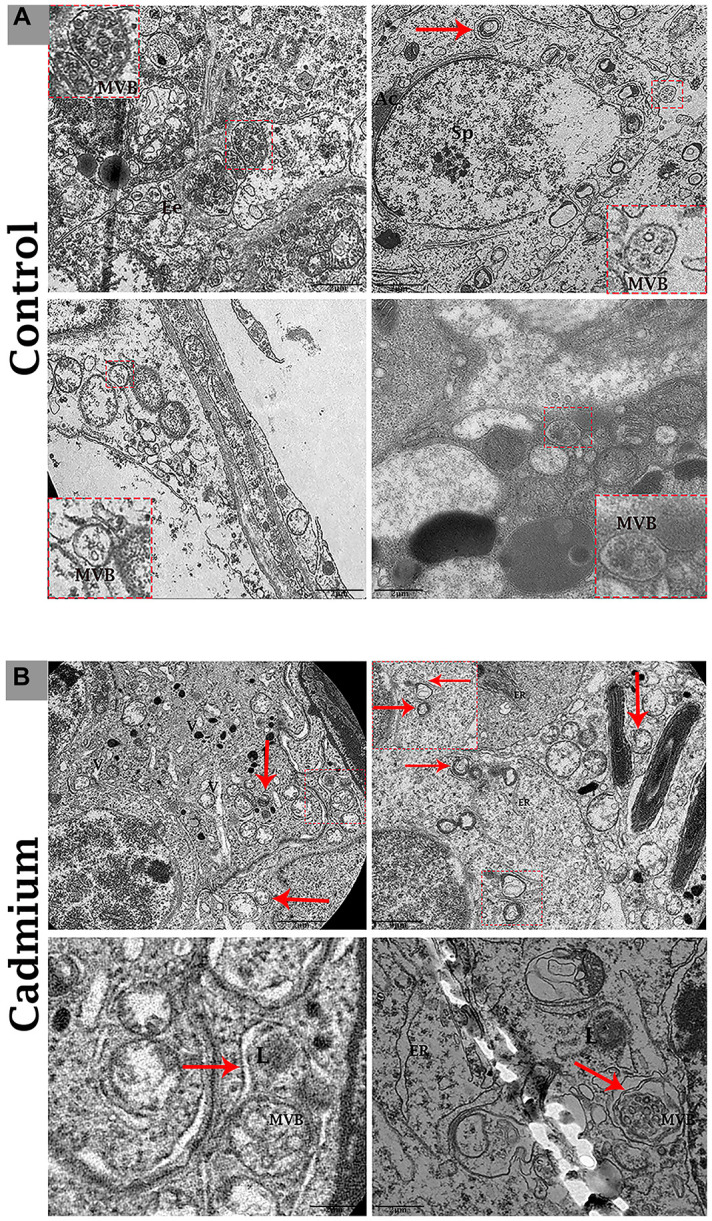
Electron micrograph of MVBs in the seminiferous tubules of control (**A**) and cadmium (**B**) treated group. (**A**) Seminiferous tubules containing several MVBs and limited formation of autophagosome. (**B**) Seminiferous tubules containing numerous formations of autophagosome and limited MVBs. Ee: early endosome; MVBs: multivesicular bodies; Ac: acromion process; Sp: spermatozoa; ER: endoplasmic reticulum; L: lysosome; (arrow) autophagosome. Scale bars 2 μm.

**Figure 4:**
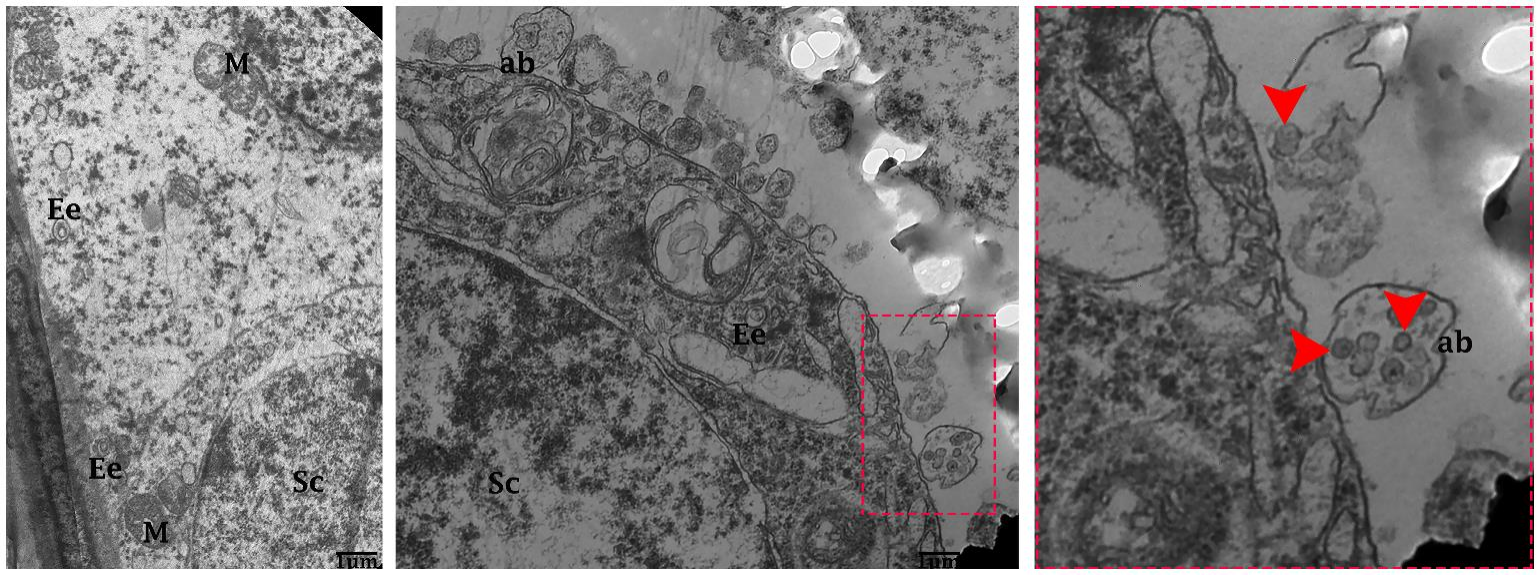
**Electron micrograph of exosomes in the seminiferous tubules of control group.** Seminiferous tubules containing formation of early endosomes and MVBs within the cytoplasmic of Sertoli cells. Apical blebs formation connected with plasma membrane of Sertoli cell. Sc: Sertoli cell; Ee: early endosome; MVBs: multivesicular bodies; M: mitochondria; ab: apical blebs; (arrow head) exosome. Scale bars 1 μm.

Transmission electron microscopy revealed that during the control group’s spermatogenesis, numerous MVBs and a limited number of autophagosomes were dispersed in the seminiferous tubules (Figure 3A). Likewise, well-developed Ee and MVBs were observed in the cytoplasm of Sertoli cells. Collectively, Sertoli cells possessed exocytic pathway, and showed some apical blebs containing various-sized nano-scale exosomes located to cell surface. Finally, apical blebs were fragmented and contents were released, and these nano-scale exosomes were directly associated with spermatozoa in luminal compartment of the seminiferous tubules of testis (Figure 4).

**Figure 5:**
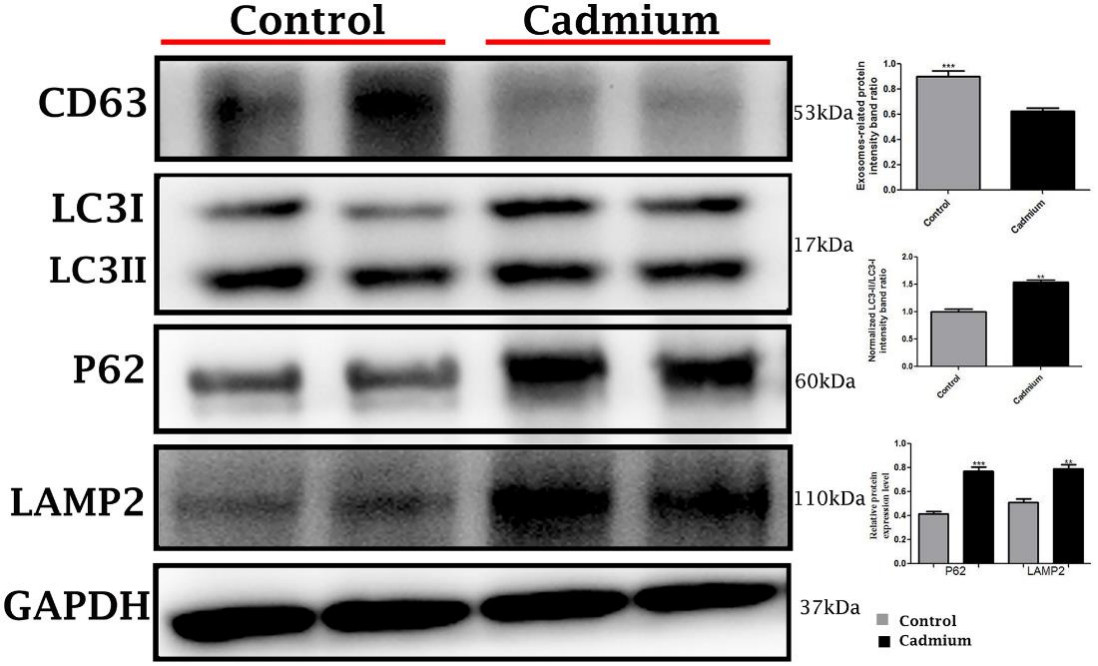
Immunoblots protein expression of autophagy-related proteins like a microtubule-associated light chain (LC3), sequestosome 1 (P62) and lysosomal-associated membrane protein 2 (LAMP2), and cluster of differentiation 63 (CD63) exosomal protein in control and cadmium treated group.

Conversely, in cadmium treated group there is very limited biogenesis of MVBs and no obvious secretions of nano-scale exosomes that interacted with spermatozoa were observed. The higher formation of autophagosomes were observed in the Sertoli cells of seminiferous tubules. Fascinatingly, the autophagosomes were enveloped by MVBs that later formed the amphisome that were degraded by lysosome (Figure 3B). As further confirmation, the immuno-blots protein expression was performed and confirmed the protein signaling of CD63 exosomal protein and autophagy-related proteins like a LC3, P62 and LAMP2. The expression level of CD63 protein was significantly reduced and LC3, P62 and LAMP2 proteins were significantly increased after cadmium treatment (Figure 5). Overall, findings demonstrated that the cadmium inhibits the secretion of nano-scale exosomes and MVBs in the Sertoli cells due to the over-activation of autophagy, which sustenance the homeostasis of spermatogenesis.

### Cadmium-induced ultrastructural modifications in testis spermatozoa

### Testis of the control group

**Figure 6:**
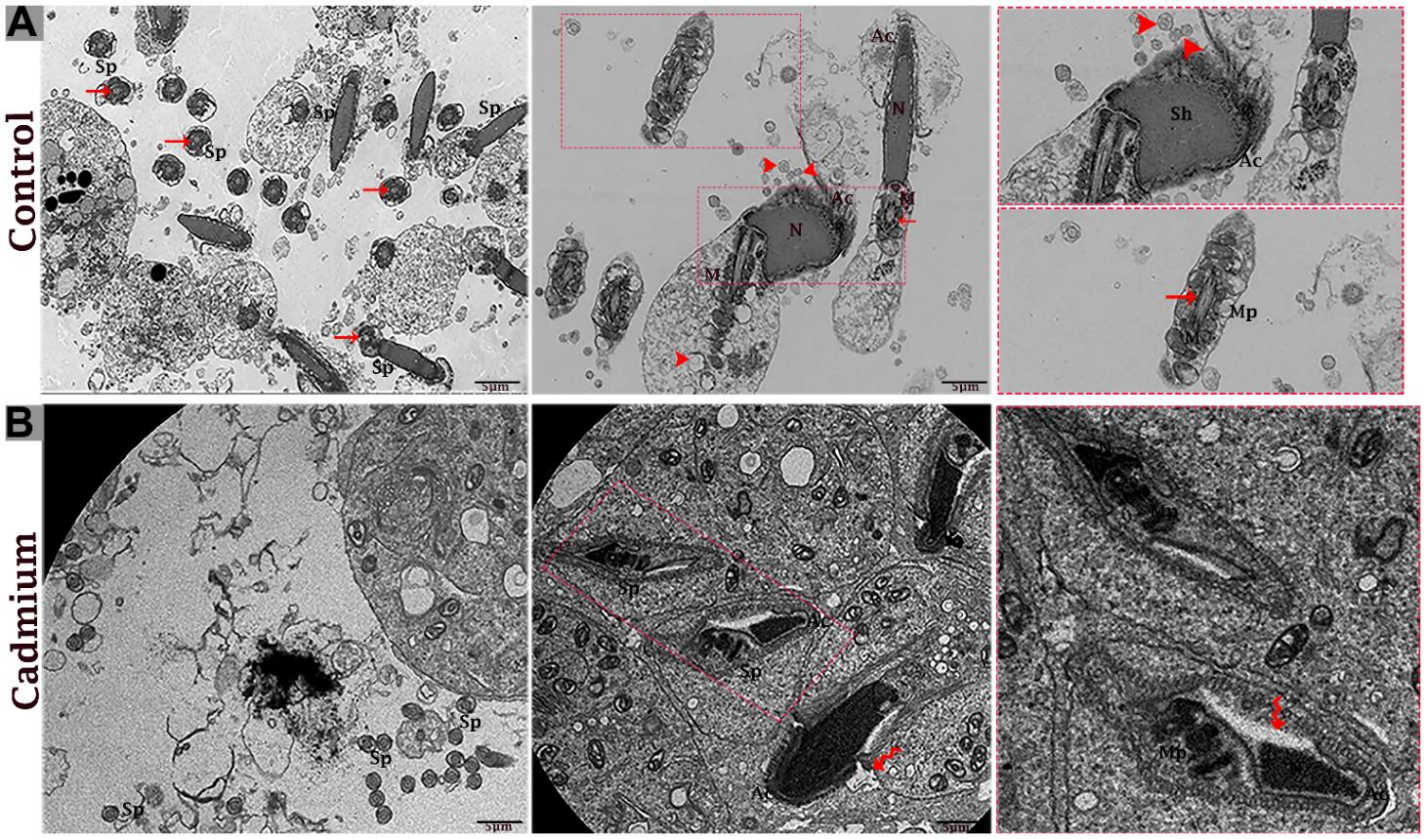
Electron micrograph of spermatozoa of control (**A**) and cadmium treated (**B**) group. (**A**) Healthy spermatozoa were observed in luminal compartment of seminiferous tubules. (**B**) Destructive spermatozoa were observed in luminal compartment of seminiferous tubules. Sp: spermatozoa; Sh: sperm head; Mp: mid-peace; N: nucleus; Ac: acrosome reaction; M: mitochondria; (arrow head) exosome; (arrow) axonema; (curved arrow) vacuolation. Scale bars 5 μm.

**Figure 7:**
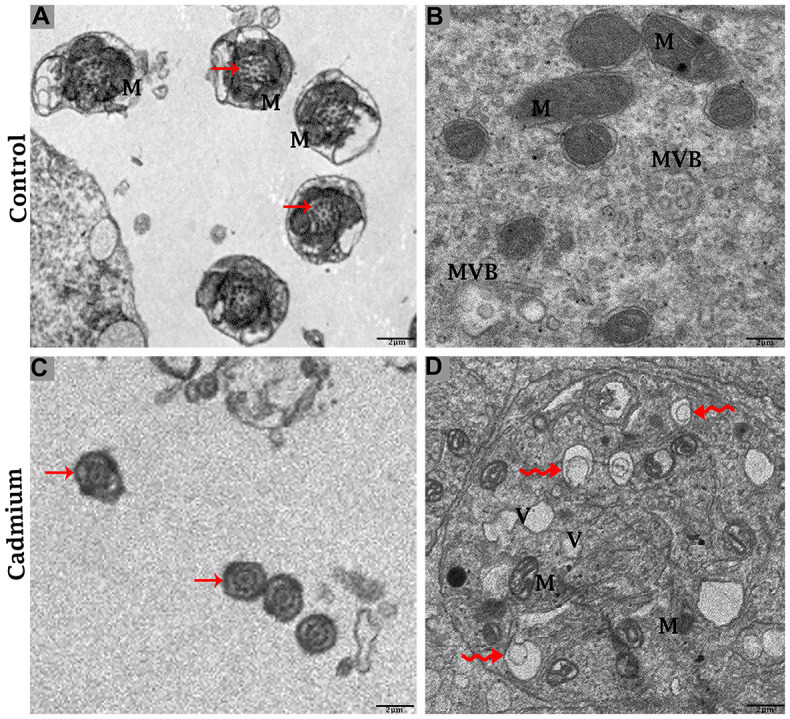
Electron micrograph of ultrastructures of mid-peace of spermatozoa and Sertoli cells of control (**A**, **B**) and cadmium treated (**C**, **D**) group. M: mitochondria; MVBs: multivesicular bodies; V: vacuole; (curved arrow) autophagosome; (arrow) axoneme. Scale bars 2 μm.

The testis of the control group was filled with healthy spermatozoa. Elongated spermatids had a well-developed acromion process that was adjacent to nano-scale exosomes. In the mid-peace of spermatids microtubules form an axoneme. In this stage numerous mitochondria were distributed throughout the mid-peace of germ cell, and closely related nano-scale exosomes were observed. In elongated spermatids tail formed the sheath of the axial filament (Figure 6A). The cytoplasm of Sertoli cells showed mitochondria were near associated with MVBs (Figure 7A, 7B).

### Testis of the cadmium treated group

The cadmium treated group’s testis was filled with limited and deformative spermatozoa with damaging acromion process, mid-peace and tail region, and vacuolization inside the cytoplasm of spermatids were observed (Figure 6B). The microtubule arrangement in the microtubular sheet that surrounded the axoneme was also disturbed. Numerous small vacuoles and degenerated mitochondria that lose their cristae matrix were observed. In addition, many autophagosomes and vacuoles were observed in the cytoplasm of Sertoli cells (Figure 7C, 7D).

## DISCUSSION

Male infertility is a developing worldwide health issue after cancer and cardio-vascular diseases [35]. Despite reports indicating an increase in male infertility and sub-fertility based on analysis of spermatozoa quality and testicular cancer [36], recent reports have confirmed that decreasing male fertility based on spermatozoa concentration and spermatozoa analysis is most likely the result of increased male exposure to environmental pollutants [37]. Cadmium has been shown to impede the male reproduction [18]. Cadmium-induced testicular destruction has been fully explored [18, 38–41]. Previous studies have observed that the testis is target organ of cadmium that damages testicular tissue and reduces spermatogenesis [42–45]. In the current study, after the cadmium treated group, the luminal compartment of seminiferous tubules showed significantly reduced number of developing spermatogonia, spermatocytes, spermatids and spermatozoa. In addition, cadmium caused the disarray of seminiferous tubules with extensive degenerative vacuolation, and more intercellular empty space in between the germ cells of the testis. Cadmium impairs testicular biochemical function and steroidogenic activity by affecting Sertoli cells and Leydig cells of the testis, as well as causing oxidative stress, cadmium induced autophagy, and germ cell apoptosis [38, 46, 47]. Cadmium exposure reduces spermatozoa quality and spermatozoa penetration power into oocytes, and disturbs the embryo development [23]. These findings are clearly linked to cadmium which represent high risk to male fertility.

Exosomes provide a new insight into intercellular communication mechanism because they provide direct evidence for the transferring of several important bio-active cargo molecules (proteins, lipids, DNA, mRNA, microRNA, circular RNA, long non-coding RNA) between testicular cells and spermatozoa [48]. In the current study, we found in the control group the numerous exosomal-MVBs secretion at the basal and luminal compartment of seminiferous tubules of testis. Exosomal secretion is well-known as essential regulator of successful reproduction and affording protection to high quality of spermatozoa, and eliminates the weak and defective spermatozoa [49, 50]. These exosomes contact with germ cells to maintain the homeostasis of spermatogenesis [51]. The exosomal cargo proteins work and perform together to promote the development of sperm motility, metabolism, oxidation reduction, acrosome reaction, capacitation, and finally fertilization [52, 53]. However, after cadmium treatment, there is very limited biogenesis of MVBs and no obvious secretion of nano-scale exosomes that interacted with spermatozoa were observed. Alterations in morphology or composition of extracellular vesicle (exosomes) may lead to reduce fertility and fetal development that has long-term effects on the health of the progeny and impair the reproductive status [14, 15]. Modified exosomal cargo proteins promote the abnormal the phonotypic and genotypic child development [54]. Although in cadmium treated group the higher formation of autophagosomes and vacuolation in spermatozoa were observed in seminiferous tubules. Fascinatingly, autophagosome was enveloped by MVBs and later formed the amphisomes, then these were degraded by lysosomes. After interruption of autophagy and MVBs pathway the target of rapamycin complex1 to be reduced under rich growing conditions [55], also over-activated autophagy which causes cell death under severe oxidative stress and metal toxicity [56]. Impeded autophagy causes abnormal development of acrosome and mitochondria crista formation during spermatogenesis [57]. Autophagy works opposite to high secretion of the exosomal-MVBs pathway [1]. Cadmium-induced autophagy impairs many body cells, and autophagic cell death can cause physiologically significant damage [18, 58]. MVBs and their contents are degraded by autophagy, and inhibiting autophagy by 3-methyaldenine may save and increase exosomes secretion from MVBs during spermatogenesis [1, 59]. These findings indicate that cadmium is capable of triggering the impairment of spermatozoa development during spermatogenesis through exosomal-MVBs pathway as mediators by changing their biogenesis and release.

## CONCLUSIONS

**Figure 8:**
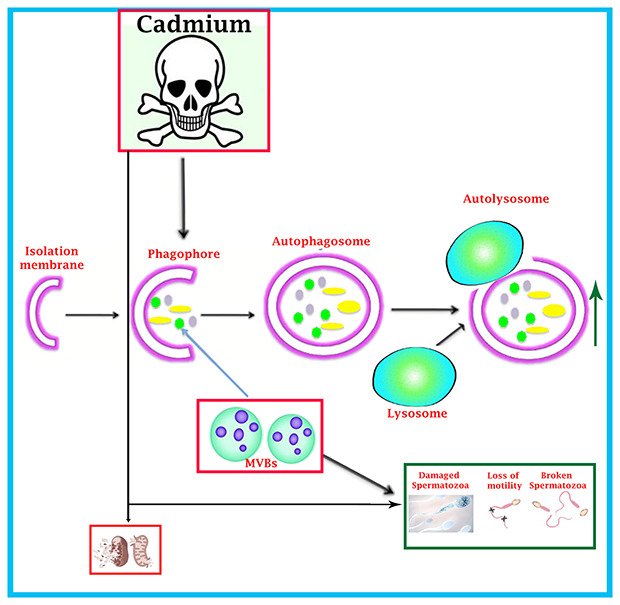
Schematic illustration of cadmium-induced more formation of autophagosomes and autolysosome that causes reduction of MVBs pathway through over activation of autophagic pathway, and spermatozoa were deprived of getting the immunological support and protection from exosomal-MVBs secretion, which leads to the destruction of spermatozoa during spermatogenesis.

In summary, we have demonstrated that cadmium has obvious toxic effect on the exocytosis pathway of exosomes-MVBs secretion, as evidenced by the inhibition of biogenesis of MVBs and exosomes secretion and over activation of the autophagic pathway in the luminal compartment of seminiferous tubules of testis, that lead to spermatozoa which were deprived from immunological defense mechanism of nano-scale exosomes and consequently, more degenerated and abnormal development of spermatozoa were observed during spermatogenesis (Figure 8). Thus, this study provides novel insight into the toxicological effect of cadmium on immunological role of nano-scale exosomes in the male reproductive system.

## MATERIALS AND METHODS

### Animals and treatment

6-week-old male C57BL/6 mice were obtained from Jiangsu University’s Experimental Animal Center (Jiangsu, China). The Yangzhou University Institutional Animal Care and Use Committee approved the study, which was carried out in accordance with the National Research Council’s Guide for the Care and Use of Laboratory Animals (approval ID: SYXK (Su) 2017- 0044). A total (n = 30) mice were randomly divided into two groups: the control group (n = 15) (which received double distilled water), and the cadmium group (n = 15) (which received 50 mg/L Cd). Water was freely available to both groups. The trial period was 12 weeks (February to April). Average body weight of each mature male C57BL/6 mice was 28.1±2.4 (control group) and 26.1±1.9 (cadmium group) After 12 weeks, all mice were anesthetized with 2% sodium pentobarbital and sacrificed through cervical dissection. The testes were quickly collected and fixed to perform the below techniques.

### Light microscopy

The testis samples were fixed overnight in 10% neutral buffered formalin before being embedded in paraffin wax. Sectioning was done at 5 μm. All such sections were stained with hematoxylin and eosin (Harry’s hematoxylin for 2 minutes and 1% eosin for 30 seconds). For light microscope analysis, an Olympus BX53 microscope and camera were used (Olympus DP73, Japan).

### Johnsen score

We used the criteria developed by Johnsen to evaluate germ cells development in the seminiferous tubules [35]. Johnsen scores use a ten-point scoring system to quantify spermatogenesis based on the cell profile encountered along the seminiferous tubules (Table 1). A Johnsen score of 10 represents the highest level of spermatogenesis activity, while a score of 1 suggests the complete absence of germ cells. We generated four distinct labels based on Johnsen scores from 1 to 10. The four labels corresponded to Johnsen scores 1-3, 4-5, 6-7, and 8-10. Johnsen scores of 1-2 contain no germ cells, a Johnsen score of 3 contains spermatogonia as germ cells, a Johnsen score of 4-5 contains spermatocytes, a Johnsen score of 6-7 contains spermatids, and a Johnsen score of 8-10 contains mature sperm. The mean score was estimated using 60 tissue sections with 2 replications of each analysis, and each group received an equal number of tissue sections (n = 30).

**Table 1:** Development of germ cells in the seminiferous tubules was classified according to the Johnsen score.

**S.No**	**Score**	
**01**	Score 10	Full spermatogenesis
**02**	Score 9	Incomplete spermatogenesis with many late spermatids
**03**	Score 8	Less than 5 spermatozoa per tubules and a few late spermatids
**04**	Score 7	No spermatozoa, but spermatids are present
**05**	Score 6	Few spermatids are present
**06**	Score 5	Only spermatocytes are present
**07**	Score 4	Few spermatocytes are present
**08**	Score 3	Spermatogonia are present
**09**	Score 2	Only Sertoli cells are present
**10**	Score 1	Just about empty lumen

It applies for the numbers from 1 to 10 to a cross-section of each tubule according to the following criteria.

### Transmission electron microscopy

For the ultrastructure analysis of testis, samples were cut into small pieces (1mm3) and fixed in 2.5% (v/v) glutaraldehyde in 0.1M phosphate-buffered saline (PBS; 4° C, pH7.4; for 24 hours). Then they were washed in the same buffer and post-fixed with 1% (w/v) osmium tetroxide for 1 hour. For the purpose of embedding the samples in araldite, the samples were dehydrated using ethanol in increasing concentrations. It was divided into sections. In order to stain the ultrathin sections (50 nm), Formvar-coated grids were used. Each staining step took 20 minutes. Transmission electron microscope was used to analyze the sections (Hitachi H-7650; Japan).

### Western blotting

The tissue samples of testis were homogenized in an ice-cold RIPA buffer containing protease and phosphate inhibitors (Roche Applied Science, Penzberg, Germany). The concentration of protein in the supernatant was measured using the bicichonic acid assay after centrifugation at 13,000 g for 15 mint, protein samples 10μl were loaded onto SDS-PAGE gels at 4-12%. Electrophoresis was performed at 120 V for 2 hr at 4° C (Bio-Red Mini-Protein), followed by immunoblotting with primary antibodies dilution (1:1000) and Actin (1:10,000) (Table 2). Targeted proteins intensities CD63, LC3, P62 and LAMP2 were normalized against actin. Western blotting quantitative measurements were performed in three independent experiments.

**Table 2:** Information for primary antibodies.

**Antibody**	**Species**	**Catalog no**	**Dilution**	**Source**
CD63	Rabbit	AB_2839529	1:1000	Affinity
LC3	Mouse	12,135-1-AP	1:1000	Proteintech
P62	Rabbit	51,145	1:1000	Cell signaling technology
LAMP2	Rabbit	66301-1-lg	1:1000	Proteintech

### Statistical analysis

All the quantification was measured through ImageJ software. The data were presented as the mean ± SEM. To determine whether there were significant differences between the two groups, a t-test was operated in GraphPad Prism. *P* < 0.05 was used to determine whether the differences were significant (one-tailed).
